# Increased MPO in Colorectal Cancer Is Associated With High Peripheral Neutrophil Counts and a Poor Prognosis: A TCGA With Propensity Score-Matched Analysis

**DOI:** 10.3389/fonc.2022.940706

**Published:** 2022-07-14

**Authors:** Meilin Weng, Ying Yue, Dan Wu, Changming Zhou, Miaomiao Guo, Caihong Sun, Qingwu Liao, Minli Sun, Di Zhou, Changhong Miao

**Affiliations:** ^1^ Department of Anesthesiology, Zhongshan Hospital, Fudan University, Shanghai, China; ^2^ Department of Anesthesiology, Shanghai Cancer Center, Fudan University, Shanghai, China; ^3^ Shanghai Key Laboratory of Perioperative Stress and Protection, Zhongshan Hospital, Fudan University, Shanghai, China; ^4^ Department of Cancer Prevention, Shanghai Cancer Center, Fudan University, Shanghai, China

**Keywords:** myeloperoxidase, colorectal cancer, preoperative neutrophil counts, prognosis, TCGA analysis, propensity score-matched analysis

## Abstract

**Background:**

Myeloperoxidase (MPO) has been demonstrated to be a local mediator of inflammation in tissue damage in various inflammatory diseases. Given its controversial effect on colorectal cancer (CRC), there has been growing interest in investigating the role of this enzyme in CRC. The mechanism underlying MPO activity and CRC progression requires further clarification.

**Methods:**

The expression and function of MPO in CRC were evaluated using TCGA analysis. TCGA, TIMER, and Human Cell Landscape analyses were used to analyze the correlation between MPO expression and neutrophil infiltration in CRC. Spearman’s bivariate correlation analysis was used to verify the correlation between MPO levels in CRC and the peripheral neutrophil count. In the clinical analysis, 8,121 patients who underwent elective surgery for CRC were enrolled in this retrospective cohort study from January 2008 to December 2014. Propensity score matching was used to address the differences in baseline characteristics. The Kaplan–Meier method and Cox regression analysis were used to identify independent prognostic factors in patients with CRC.

**Results:**

MPO was upregulated in CRC tissues, which is related to malignant progression and worse survival in CRC patients from TCGA analysis. MPO was significantly correlated with the infiltration level of neutrophils in CRC in TCGA, TIMER, and Human Cell Landscape analyses. MPO was positively correlated with the peripheral neutrophil count. Data of the 8,121 patients who underwent CRC surgery were available for analysis. After propensity score matching, 3,358 patients were included in each group. Kaplan–Meier survival curves showed that high preoperative neutrophil levels were associated with decreased overall survival (OS; P < 0.001) and disease-free survival (DFS; P = 0.015). The preoperative neutrophil count was an independent risk factor for OS (hazard ratio [HR], 1.157; 95% confidence interval [CI], 1.055–1.268; P = 0.002) and DFS (HR, 1.118; 95% CI, 1.009–1.238; P = 0.033).

**Conclusions:**

Our research indicates that increased MPO levels in CRC are significantly correlated with high preoperative neutrophil counts, and both serve as prognostic indicators for worse survival in CRC patients. Our study suggests that neutrophils may be key players in the mechanism linking MPO levels with poor CRC outcomes.

## Introduction

Colorectal cancer (CRC) ranks third and second in terms of morbidity and mortality, respectively, among the various cancer types worldwide (1). In China, although CRC ranks fifth as the main cause of cancer-associated death among cancer patients, the mortality accompanying this malignancy has been on the rise in the past few decades (2, 3). Currently, the most common treatment for CRC patients is surgical resection; however, approximately half of the patients relapse within three years after surgery (4). Thus, a prognostic indicator or potential therapeutic target is urgently needed for predicting survival outcomes in CRC patients.

Myeloperoxidase (MPO), a member of the heme peroxidase superfamily, plays a key role in regulating the functions of neutrophils and monocytes (5). MPO is mainly involved in the formation of reactive oxygen species or hypochlorous acid, thus resulting in tissue damage (6–8). An association between MPO and disease has been reported in ovarian and cervical cancers, as well as in CRCs (9, 10). Some studies showed that high preoperative MPO levels improved prognosis in CRC (11, 12), while others reported that MPO promoted malignant phenotypes in CRC patients (13, 14). Given the controversial effect of MPO on CRC, there has been growing interest in investigating the role of this enzyme in CRC. MPO is the most abundant protein expressed by neutrophils and it may also have the greatest potential to damage living cells (15). In autoimmune diseases, MPO exists only in the cytoplasm of the neutrophils (16). Therefore, we were curious about the relationship between MPO levels in CRC and peripheral neutrophil counts.

CRC is a highly heterogeneous tumor, which is closely associated with inflammation and characterized by the infiltration of various immune cells (17). Various peripheral inflammatory markers such as neutrophil and lymphocyte counts are easy to obtain from conventional preoperative laboratory examinations (18, 19). The correlation between a high white blood cell (WBC) count and poor prognosis has been identified in various cancer types, such as oropharyngeal cancer, cervical cancer, and esophageal cancer (20–22). Neutrophils, which are crucial regulators of both inflammation and immune responses, account for 50–70% of leukocytes in circulation and are the major elements of WBCs (23). However, studies on the correlation between preoperative neutrophil count and the prognosis of CRC are controversial. Most evidence shows that neutrophils can promote tumors, and the degree of neutrophil infiltration is related to poor prognosis; however, a few studies have collected evidence that neutrophils can either improve or exert no effect on prognosis (24, 25). We aimed to verify the function of preoperative neutrophils in a large sample cohort. The underlying mechanism behind MPO and the prognosis of patients with CRC have not yet been clarified.

This study aimed to assess the expression and function of MPO in CRC using TCGA analysis and to analyze the potential correlations between MPO in CRC and peripheral neutrophil counts. We further verified the prognostic value of preoperative neutrophil counts for OS and DFS after CRC surgery in our large sample cohort. We speculated that increased MPO levels in CRC were positively correlated with high preoperative peripheral neutrophil counts, both of which predicted worse survival outcomes in CRC patients undergoing elective surgery.

## Materials and Methods

### RNA-Sequencing Data and Bioinformatics Analysis

Gene expression data with clinical information from Colon adenocarcinoma (COAD) patients (521 cases, workflow type: HTSeq-TPM) were collected from TCGA using the R package “TCGAbiolinks". The exclusion criteria were normal colorectal samples and an OS of < 30 days. The TPM data from 521 cases were used for further analyses. Of the 521 samples, 480 were tumor tissues and 41 were normal tissues. Normal tissue is the tissue adjacent to a tumor, specifically at a distance of 2 cm from the tumor. Patient characteristics including sex, age, BMI, TNM stage, pathological stage, primary therapy outcome, residual tumor, CEA level, perineural invasion, lymphatic invasion, history of colon polyps, presence of colon polyps, OS event, disease-specific survival (DSS) event, and progression-free interval (PFI) event were recorded. Unavailable or unknown clinical features were considered missing values. This study met the TCGA publication guidelines. All data used in the study were obtained from TCGA.

### Immune Infiltration Analysis Using ssGSEA

Immune infiltration analysis of CRC was performed using the single-sample Gene Set Enrichment Analysis (ssGSEA) method using the GSVA package in R (version 3.6.3) for 24 types of immune cells in tumor samples, including neutrophils, mast cells, eosinophils, macrophages, natural killer (NK) cells, CD56dim NK cells, CD56bright NK cells, dendritic cells (DCs), immature DCs (iDCs), activated DCs (aDCs), plasmacytoid DCs (pDCs), T cells, CD8+ T cells, T helper cells, Th1 cells, Th2 cells, Th17 cells, T follicular helper cells (Tfhs), Tregs, effector memory T cells (Tems), central memory CD4+ T cells (Tcms), γδT cells (Tgd), cytotoxic cells, and B cells. The correlation between MPO and these immune cells was analyzed using the Spearman correlation test, and the infiltration of immune cells between the high and low MPO-expression groups was analyzed using the Wilcoxon rank-sum test.

#### GEPIA2

GEPIA2 is a website developed by Zhang Zemin’s laboratory at Peking University. It can analyze the RNA-seq expression data of 9736 tumor samples and 8587 normal samples from TCGA and GTEx projects (26).

#### OncoLnc

Using OncoLnc (OncoLnc), we collected the survival data of 8647 patients with 21 kinds of tumors from TCGA and the corresponding mRNA and miRNA expression profile data. Simultaneously, the lncRNA expression data from the MiTranscriptome project were collected to perform survival analysis which can be easily used to explore survival-related genes in various tumors.

### TIMER

TIMER (http://cistrome.org/TIMER/) was used to systematically analyze the infiltration of immune cells in different types of cancer. The abundance of six types of immunoreactive substances (B cells, CD4^+^ T cells, CD8^+^ T cells, neutrophils, macrophages, and DCs) was estimated using the TIMER algorithm. The partial correlation coefficient indicates the relationship between variables. A partial correlation coefficient greater than 0.7 implies a very close relationship; that in the range 0.4–0.7 indicates a close relationship; that in the range 0.2–0.4 indicates a moderate relationship; and that lower than 0.2 indicates a distant relationship.

### Human Cell Landscape

The Human Cell Landscape database (http://bis.zju.edu.cn/HCL/) is a public single-cell RNA sequencing database that contains the cell type composition of major human organs and a basic scheme for the Human Cell Landscape. The evaluation of the relationship between MPO and neutrophils was performed using the data analyzed in this database.

### Immunohistochemical Staining

Paraffin-embedded tissues were stained with an MPO antibody (ab208670, Abcam, Cambridge, UK). The staining score was determined by two experienced pathologists at the Zhongshan Hospital (China). Six high-power fields (HPFs; ×200 magnification) were randomly counted by the two independent pathologists (each with three fields). The IHC score ranged from 0 to 300, according to the sum of the percentage of stained cells.

### RNA Separation and Real-Time Quantitative PCR

Total RNA was extracted using the TRIzol reagent (Invitrogen, Waltham, MA, USA). cDNA was obtained by reverse transcription using the PrimeScript RT kit (Takara, Shiga, Japan). The expression of candidate genes and the housekeeping gene GAPDH was evaluated *via* quantitative real-time PCR using the ABI 7900HT real-time PCR system (Applied Biosystems, Carlsbad, CA, USA). The relative transcription levels were calculated using the 2−ΔΔCT method. GAPDH (human) primer sequences: 5’-GGAGCGAGATCCCTCCAAAAT-3’; 5’-GGCTGTTGTCATACTTCTCATGG-3’. MPO (human) primer sequences: 5’-TGCTGCCCTTTGACAACCTG-3’; 5’-TGCTCCCGAAGTAAGAGGGT-3’.

### Clinical Study Design

This clinical retrospective study was performed at the Shanghai Cancer Center, Fudan University, Shanghai, China. The study was approved by the center’s Ethics Committee (IRB2105235-6) and informed consent was obtained from all subjects involved in the study.

### Clinical Study Population and Data Sources

Of the 13,721 patients who underwent elective surgery for CRC between January 2008 and December 2014, a total of 8,121 with clinical features and survival data were included in this study. The inclusion criteria were as follows: CRC diagnosed by histological evidence, patients undergoing elective radical surgery for CRC, and patients older than 20 years of age. We excluded patients with incomplete medical records, benign tumors or carcinomas in situ, emergency operations, an ASA physical status score > 3, metastasis at initial visit and a history of malignant tumors. Sixty-nine pairs of CRC tissues and their matched adjacent non-cancerous tissues from 8,121 patients were used for IHC and qPCR analyses. These 69 patients underwent elective surgery for CRC in December 2014.

Patients were divided into two groups according to their preoperative neutrophil counts. Those with preoperative neutrophils > 3.5×10^9^/L were defined as the high preoperative neutrophil group. The cut-off value for neutrophils was calculated using the receiver operating characteristic (ROC) curve; the threshold was associated with an increased risk of postoperative mortality and was within the normal range of the neutrophil count.

### Variables and Outcomes

We reviewed and recorded the following data from the clinical information system of the Shanghai Cancer Center: sex, age, preoperative adjuvant chemotherapy, surgical approach, tumor histology, tumor differentiation, surgical margin positivity, TNM stage, infiltrating lymph nodes > 12, number of cancer nodules > 1, surgery again within 30 days, death, intraoperative transfusion, and blood loss.

The primary endpoints were OS and DFS; OS was defined as the interval between the date of diagnosis and the date of death for any reason, while DFS was defined as the interval between the date of diagnosis and the date of recurrence, metastasis, secondary primary tumor, or death.

The relationship between the Mismatch Repaire-status and neutrophil counts, and the effect of MMR-status on survival in 668 patients with CRC.

We analyzed 668 patients with MMR-status. The chi-squared test was used to evaluate the differences in MMR-status between high/low preoperative neutrophil counts. Kaplan-Meier method was used to evaluate the overall survival differences between MMR-proficient and MMR-deficient patients.

### Statistical Analyses

In TCGA analysis, all statistical analyses were conducted and plots were generated using the R software version 3.4.4 (R Foundation for Statistical Computing, Austria). The Wilcoxon rank-sum and Wilcoxon signed-rank tests were used to analyze the expression of MPO in non-paired and paired samples, respectively. The Kruskal–Wallis test, Wilcoxon signed-rank test, and logistic regression were used to evaluate the relationships between clinicopathological features and MPO expression. The median MPO expression level was regarded as the cut-off value. Cox regression analyses and the Kaplan–Meier method were used to evaluate prognostic factors. Accordingly, a univariate Cox analysis was used to compare the effect of MPO expression on survival and other clinical features. An ROC curve was used to further evaluate the value of the biomarker, and nomograms were constructed to predict the 1-, 3-, and 5-year survival probabilities. Spearman’s correlation and Wilcoxon signed-rank tests were used to analyze the correlation between MPO expression and neutrophil counts. In correlation analyses, the correlation coefficient indicates the relationship between variables. A correlation coefficient above 0.7 shows that the relationship is very close; that in the range 0.4–0.7 shows a moderate relationship; and that in the range 0.2–0.4 shows a low correlation.

In the clinical study, analyses were performed using IBM SPSS Statistics 25.0 (SPSS Corp., Armonk, NY, USA). The chi-squared test was used to evaluate the differences in baseline patient characteristics between the two groups. To reduce possible confounding factors, propensity score matching was performed. The key confounders including sex, preoperative adjuvant chemotherapy, tumor differentiation, tumor histology, surgical margin positive, lymph node invasion > 12, and number of cancer nodule ≥ 1 were matched. We used the R package “MatchIt” for propensity score matching.

In the propensity-matched cohort, the Kaplan–Meier method was used to compare OS and DFS using the log-rank test. Cox proportional hazards models were used to confirm the independent prognostic factors for CRC patients. All variables were adjusted using a univariate Cox proportional hazards model. Variables with P < 0.05 were included in the multivariate analysis. A multivariate Cox proportional hazards model was used in a stepwise manner to select the prognostic factors. The hazard ratio (HR) and corresponding 95% confidence interval (CI) were calculated. In all tests, P-values < 0.05 were considered statistically significant. Differences were considered significant at * P < 0.05, ** P < 0.01, and *** P < 0.001.

## Results

### TCGA Analysis: MPO Is Upregulated in CRC Tissues

To analyze the relationship between MPO expression and CRC, the MPO expression data and detailed clinical characteristics of 478 CRC patients were downloaded from TCGA, including TNM stage, pathological stage, primary therapy outcome, sex, age, BMI, residual tumor, CEA level, perineural invasion, lymphatic invasion, history of colon polyps, presence of colon polyps, and mortality (**Table 1**).

**Table 1:** The relationship between MPO expression and the clinicopathological features of CRC in TCGA.

Characteristic	Low expression of MPO	High expression of MPO	p
n	239	239	
T stage, n (%)			0.208
T1	6 (1.3%)	5 (1%)	
T2	48 (10.1%)	35 (7.3%)	
T3	160 (33.5%)	163 (34.2%)	
T4	24 (5%)	36 (7.5%)	
N stage, n (%)			0.739
N0	146 (30.5%)	138 (28.9%)	
N1	51 (10.7%)	57 (11.9%)	
N2	42 (8.8%)	44 (9.2%)	
M stage, n (%)			0.004
M0	182 (43.9%)	167 (40.2%)	
M1	21 (5.1%)	45 (10.8%)	
Pathological stage, n (%)			0.003
Stage I	49 (10.5%)	32 (6.9%)	
Stage II	92 (19.7%)	95 (20.3%)	
Stage III	74 (15.8%)	59 (12.6%)	
Stage IV	21 (4.5%)	45 (9.6%)	
Primary therapy outcome, n (%)			0.402
PD	10 (4%)	15 (6%)	
SD	2 (0.8%)	2 (0.8%)	
PR	5 (2%)	8 (3.2%)	
CR	113 (45.2%)	95 (38%)	
Sex, n (%)			0.234
Female	120 (25.1%)	106 (22.2%)	
Male	119 (24.9%)	133 (27.8%)	
Age, n (%)			0.926
<=65	96 (20.1%)	98 (20.5%)	
>65	143 (29.9%)	141 (29.5%)	
BMI, n (%)			0.724
<25	42 (16.4%)	45 (17.6%)	
>=25	87 (34%)	82 (32%)	
Residual tumor, n (%)			0.215
R0	172 (46%)	174 (46.5%)	
R1	3 (0.8%)	1 (0.3%)	
R2	8 (2.1%)	16 (4.3%)	
CEA level, n (%)			0.010
<=5	107 (35.3%)	89 (29.4%)	
>5	41 (13.5%)	66 (21.8%)	
Perineural invasion, n (%)			0.372
NO	68 (37.6%)	67 (37%)	
YES	19 (10.5%)	27 (14.9%)	
Lymphatic invasion, n (%)			0.054
NO	139 (32%)	127 (29.3%)	
YES	71 (16.4%)	97 (22.4%)	
History of colon polyps, n (%)			0.989
NO	133 (32.6%)	129 (31.6%)	
YES	75 (18.4%)	71 (17.4%)	
Colon polyps present, n (%)			0.963
NO	82 (32.9%)	80 (32.1%)	
YES	43 (17.3%)	44 (17.7%)	
OS event, n (%)			0.045
Alive	197 (41.2%)	178 (37.2%)	
Dead	42 (8.8%)	61 (12.8%)	
DSS event, n (%)			0.011
Alive	209 (45.2%)	189 (40.9%)	
Dead	22 (4.8%)	42 (9.1%)	
PFI event, n (%)			0.005
Alive	189 (39.5%)	161 (33.7%)	
Dead	50 (10.5%)	78 (16.3%)	

**Figure 1:**
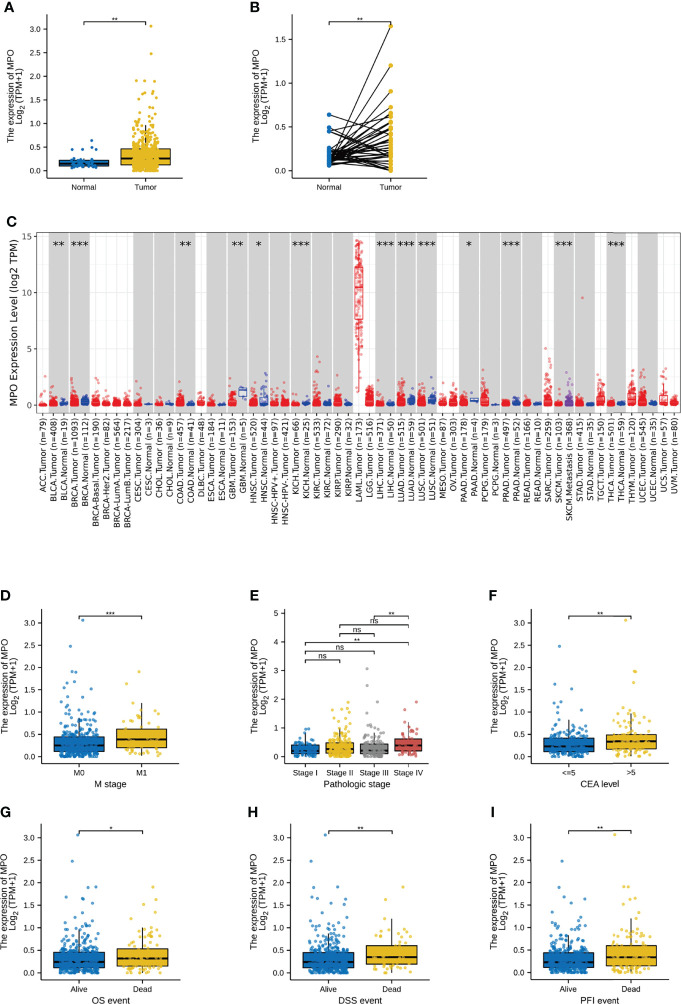
MPO was upregulated in CRC and correlated with the malignant progression of CRC patients. **(A)** The MPO expression levels in 480 tumor tissues and 41 normal tissues. **(B)** The expression of MPO in 41 normal and matched tumor tissues. **(C)** MPO expression in several cancers in the TIMER2 database. **(D–F)** The association with elevated MPO and clinicopathological characteristics, including M stages, pathological stage, and CEA level. **(G–I)** The association with elevated MPO and death events, including OS events, DSS events, and PFI events. The difference was considered significant at * P < 0.05, ** P < 0.01, or *** P < 0.001. ns, no significance.

We used the Wilcoxon signed-rank test to compare the expression of MPO in CRC tissues and normal tissues in TCGA. MPO expression levels in 480 tumor tissues were markedly higher than those in 41 normal tissues (P = 0.001; **Figure 1A**). Correspondingly, analysis of MPO expression in 41 paired CRC tissues and their matched non-cancerous tissues also showed a significant upregulation of MPO in patients with CRC (P = 0.002; **Figure 1B**). MPO is also highly expressed in certain cancers, such as colon adenocarcinoma, pancreatic adenocarcinoma, and acute myeloid leukemia, as inferred from the TIMER2 database (**Figure 1C**).

### TCGA Analysis: The Upregulation of MPO Is Related to the Malignant Progression of CRC Patients

Under the Kruskal–Wallis test and Wilcoxon signed-rank test in our current study, a higher level of MPO expression was significantly correlated with a higher M stage (P = 0.001), a higher pathological stage (stage I vs. IV, P = 0.001; stage III vs. IV, P = 0.002), and a higher CEA level (P = 0.005; **Figures 1D–F**). Furthermore, higher MPO expression was associated with higher mortality in terms of OS (P = 0.041), DSS (P = 0.005), and PFI (P = 0.004) (**Figures 1G–I**). However, the comparison of MPO expression in the patient characteristics including T stage, N stage, history of colon polyps, presence of colon polyps, lymphatic invasion, perineural invasion, residual tumor, BMI, age, and sex was not statistically significant (P > 0.05; **Supplementary Figures 1A–J**).

To further analyze the role of MPO in CRC, we divided the patients into two groups based on MPO expression. The median expression level was used as the cut-off point for grouping. As shown in **Table 1**, high expression of MPO was strongly associated with a more advanced M stage (P = 0.004), higher pathological stage (P = 0.003), higher CEA level (P = 0.010), more OS death events (P = 0.045), more DSS death events (P = 0.011), and more PFI death events (P = 0.005); in contrast, it was not associated with the T stage, N stage, primary therapy outcome, sex, age, BMI, residual tumor, perineural invasion, lymphatic invasion, history of colon polyps, or colon polyps present (all P > 0.05) (**Table 1**).

Importantly, logistic regression analysis of MPO expression and clinicopathological features also confirmed the relationship between highly expressed MPO and the pathological stage (OR: 1.479; 95% CI: 1.024–2.141; P = 0.037 for stage III & IV vs. stage I & II, respectively), age (OR: 0.636; 95% CI: 0.439–0.917; P = 0.016 for > 65 vs. ≤ 65), and lymphatic invasion (OR: 1.495; 95% CI: 1.015–2.208; P = 0.042 for Yes vs. No); see **Table 2**. Together, our evaluation revealed that high MPO expression is related to the malignant progression of CRC.

**Table 2:** The relationship between increased MPO expression and clinicopathological features by logistic regression.

Characteristics	Total (N)	Odds Ratio (OR)	P value
T stage (T3&T4 vs. T1&T2)	477	0.848 (0.539-1.333)	0.476
N stage (N1&N2 vs. N0)	478	1.321 (0.916-1.907)	0.136
M stage (M1 vs. M0)	415	1.531 (0.903-2.623)	0.116
Pathological stage (Stage III&Stage IV vs. Stage I&Stage II)	467	1.479 (1.024-2.141)	0.037
Sex (Male vs. Female)	478	0.874 (0.610-1.252)	0.464
Age (>65 vs. <=65)	478	0.636 (0.439-0.917)	0.016
BMI (>=25 vs. <25)	256	1.115 (0.663-1.874)	0.680
Residual tumor (R1&R2 vs. R0)	374	1.864 (0.851-4.305)	0.128
CEA level (>5 vs. <=5)	303	0.929 (0.579-1.492)	0.760
Perineural invasion (YES vs. NO)	181	1.604 (0.804-3.311)	0.188
Lymphatic invasion (YES vs. NO)	434	1.495 (1.015-2.208)	0.042
History of colon polyps (YES vs. NO)	408	0.767 (0.510-1.150)	0.200
Colon polyps present (YES vs. NO)	249	1.165 (0.690-1.977)	0.569

### TCGA Analysis: The Upregulation of MPO Is Related to Worse Survival in CRC Patients

**Figure 2:**
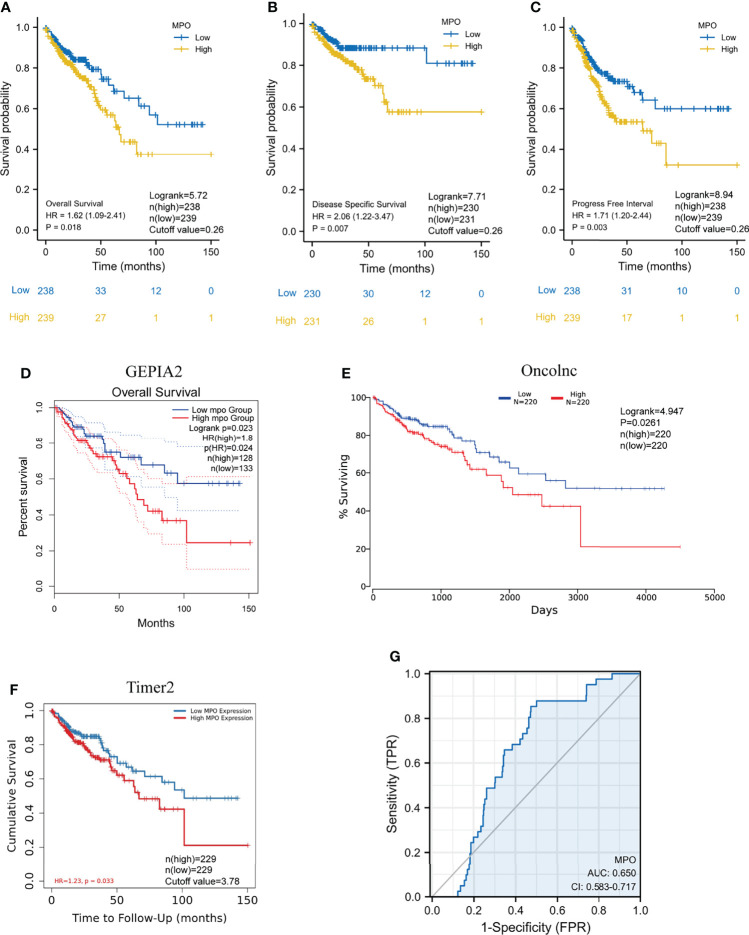
High expression of MPO is related to poor prognosis in CRC patients. **(A–C)** Kaplan–Meier analyses of OS, PFI, and DSS between the low- and high-MPO groups in TCGA. OS: Logrank=5.72, P=0.018, n(high)=238, n(low)=239, cutoff value=0.26; DSS: Logrank=7.71, P=0.007, n(high)=230, n(low)=231, cutoff value=0.26; PFI: Logrank=8.94, P=0.003, n(high)=238, n(low)=239, cutoff value=0.26. **(D–F)** Gene Expression Profiling Interactive Analysis2 (GEPIA2), OncoLnc, and TIMER2 databases were used to analyze overall survival between high and low MPO groups, respectively. GEPIA2 OS: P=0.023, n(high)=128, n(low)=133; OncoLnc OS: Logrank=4.947, P=0.0261, n(high)=220, n(low)=220, cutoff value=3.78; TIMER2 OS: P=0.033, n(high)=229, n(low)=229. **(G)** ROC analysis with respect to the MPO expression.

To gain a deeper insight into the correlation between MPO expression and the prognosis of CRC patients in TCGA, Kaplan–Meier survival analyses were conducted for OS, PFI, and DSS events in CRC patients. We observed that high MPO expression was associated with a shorter OS (HR: 1.62; 95% CI: 1.09–2.41; P = 0.018), worse DSS (HR: 2.06; 95% CI: 1.22–3.47; P = 0.007) and poorer PFI (HR: 1.71; 95% CI: 1.20–2.44; P = 0.003); see **Figures 2A–C**. This suggests that a higher expression of MPO is related to worse survival in CRC patients. We also used the Gene Expression Profiling Interactive Analysis2 (GEPIA2), OncoLnc, and TIMER2 databases to analyze TCGA data sets. The results of the survival analysis performed using these three database showed that a high expression of MPO is associated with worse prognosis (**Figures 2D–F**).

**Figure 3:**
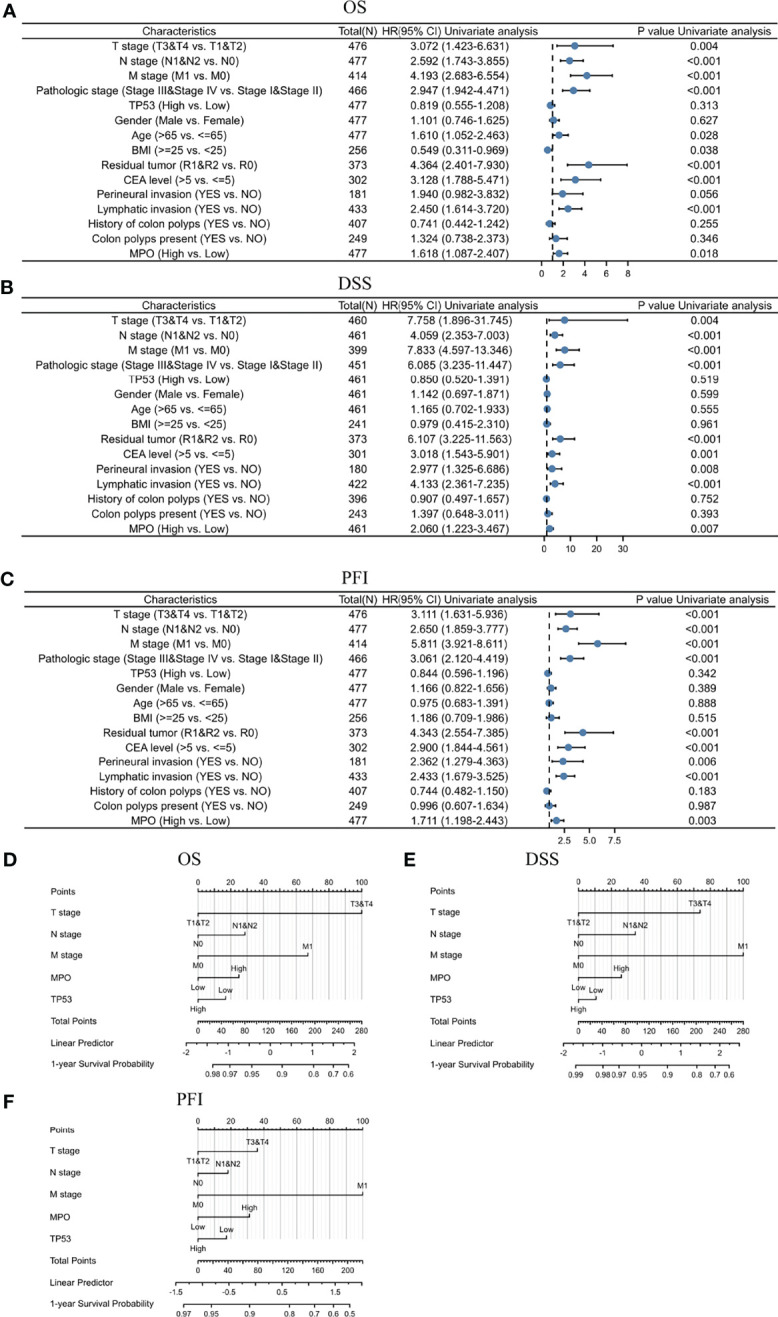
Univariate analysis of MPO for survival using the Cox regression model and nomogram based on MPO and clinical variables for survival in CRC. **(A)** Univariate analysis of prognostic factors for OS using the Cox regression model. **(B)** Univariate analysis of prognostic factors for DSS using the Cox regression model. **(C)** Univariate analysis of prognostic factors for PFI using the Cox regression model. **(D)** Nomogram for OS prognosis in CRC. **(E)** Nomogram for DSS prognosis in CRC. **(F)** Nomogram for PFI prognosis in CRC.

Moreover, we performed a univariate analysis of prognostic factors for OS using the Cox regression model (**Table 3** and **Figure 3A**). High MPO expression was associated with worse OS (HR, 1.618; CI: 1.087–2.407; P = 0.018). In addition, a higher T stage (HR, 3.072; CI: 1.423–6.631, P = 0.004), higher N stage (HR, 2.592; CI: 1.743–3.855; P < 0.001), higher M stage (HR, 4.193; 95% CI: 2.683–6.554; P < 0.001), higher pathological stage (HR, 2.947; 95% CI: 1.942–4.471; P < 0.001), older age (HR, 1.610; 95% CI: 1.052–2.463; P = 0.028), higher BMI (HR, 0.549; 95% CI: 0.311–0.969; P = 0.038), residual tumor (HR, 4.364; 95% CI: 2.401–7.930; P < 0.001), higher CEA level (HR, 3.128; 95% CI: 1.788–5.471; P < 0.001) and lymphatic invasion (HR, 2.450; 95% CI: 1.614–3.720; P < 0.001) were also associated with poor OS (**Table 3**). We also conducted univariate analyses for DSS and PFI using the Cox regression model (**Tables 4**, **5**; **Figures 3B, C**). Similarly, MPO levels were correlated with poorer PFI (HR, 1.711; 95% CI: 1.198–2.443; P = 0.003) and DSS (HR, 2.060; 95% CI: 1.223–3.467; P = 0.007). In summary, MPO is an independent risk factor for OS, PFI, and DSS in patients with CRC.

**Table 3:** Univariate analysis of prognostic factors for OS with the Cox regression model.

Characteristics	Total (N)	Univariate analysis HR (95% CI)	Univariate analysis P value
T stage (T3&T4 vs. T1&T2)	476	3.072 (1.423-6.631)	0.004
N stage (N1&N2 vs. N0)	477	2.592 (1.743-3.855)	<0.001
M stage (M1 vs. M0)	414	4.193 (2.683-6.554)	<0.001
Pathological stage (Stage III&Stage IV vs. Stage I&Stage II)	466	2.947 (1.942-4.471)	<0.001
TP53 (High vs. Low)	477	0.819 (0.555-1.208)	0.313
Sex (Male vs. Female)	477	1.101 (0.746-1.625)	0.627
Age (>65 vs. <=65)	477	1.610 (1.052-2.463)	0.028
BMI (>=25 vs. <25)	256	0.549 (0.311-0.969)	0.038
Residual tumor (R1&R2 vs. R0)	373	4.364 (2.401-7.930)	<0.001
CEA level (>5 vs. <=5)	302	3.128 (1.788-5.471)	<0.001
Perineural invasion (YES vs. NO)	181	1.940 (0.982-3.832)	0.056
Lymphatic invasion (YES vs. NO)	433	2.450 (1.614-3.720)	<0.001
History of colon polyps (YES vs. NO)	407	0.741 (0.442-1.242)	0.255
Colon polyps present (YES vs. NO)	249	1.324 (0.738-2.373)	0.346
MPO (High vs. Low)	477	1.618 (1.087-2.407)	0.018

**Table 4:** Univariate analysis of prognostic factors for DSS with the Cox regression model.

Characteristics	Total (N)	Univariate analysis HR (95% CI)	Univariate analysis P value
T stage (T3&T4 vs. T1&T2)	460	7.758 (1.896-31.745)	0.004
N stage (N1&N2 vs. N0)	461	4.059 (2.353-7.003)	<0.001
M stage (M1 vs. M0)	399	7.833 (4.597-13.346)	<0.001
Pathological stage (Stage III&Stage IV vs. Stage I&Stage II)	451	6.085 (3.235-11.447)	<0.001
TP53 (High vs. Low)	461	0.850 (0.520-1.391)	0.519
Sex (Male vs. Female)	461	1.142 (0.697-1.871)	0.599
Age (>65 vs. <=65)	461	1.165 (0.702-1.933)	0.555
BMI (>=25 vs. <25)	241	0.979 (0.415-2.310)	0.961
Residual tumor (R1&R2 vs. R0)	373	6.107 (3.225-11.563)	<0.001
CEA level (>5 vs. <=5)	301	3.018 (1.543-5.901)	0.001
Perineural invasion (YES vs. NO)	180	2.977 (1.325-6.686)	0.008
Lymphatic invasion (YES vs. NO)	422	4.133 (2.361-7.235)	<0.001
History of colon polyps (YES vs. NO)	396	0.907 (0.497-1.657)	0.752
Colon polyps present (YES vs. NO)	243	1.397 (0.648-3.011)	0.393
MPO (High vs. Low)	461	2.060 (1.223-3.467)	0.007

**Table 5:** Univariate analysis of prognostic factors for PFI with the Cox regression model.

Characteristics	Total (N)	Univariate analysis HR (95% CI)	Univariate analysis P value
T stage (T3&T4 vs. T1&T2)	476	3.111 (1.631-5.936)	<0.001
N stage (N1&N2 vs. N0)	477	2.650 (1.859-3.777)	<0.001
M stage (M1 vs. M0)	414	5.811 (3.921-8.611)	<0.001
Pathological stage (Stage III&Stage IV vs. Stage I&Stage II)	466	3.061 (2.120-4.419)	<0.001
TP53 (High vs. Low)	477	0.844 (0.596-1.196)	0.342
Sex (Male vs. Female)	477	1.166 (0.822-1.656)	0.389
Age (>65 vs. <=65)	477	0.975 (0.683-1.391)	0.888
BMI (>=25 vs. <25)	256	1.186 (0.709-1.986)	0.515
Residual tumor (R1&R2 vs. R0)	373	4.343 (2.554-7.385)	<0.001
CEA level (>5 vs. <=5)	302	2.900 (1.844-4.561)	<0.001
Perineural invasion (YES vs. NO)	181	2.362 (1.279-4.363)	0.006
Lymphatic invasion (YES vs. NO)	433	2.433 (1.679-3.525)	<0.001
History of colon polyps (YES vs. NO)	407	0.744 (0.482-1.150)	0.183
Colon polyps present (YES vs. NO)	249	0.996 (0.607-1.634)	0.987
MPO (High vs. Low)	477	1.711 (1.198-2.443)	0.003

In addition, an ROC curve was generated to further evaluate the value of MPO as a biomarker for CRC (**Figure 2G**). MPO exhibited a good predictive ability in patients with CRC (AUC: 0.650; CI: 0.584–0.717). By combining the expression levels of MPO and clinical variables, nomograms were constructed to predict the 1-, 3-, and 5-year survival (OS, PFI, and DSS) probability of patients (**Figures 3D–F**). Overall, MPO has a good predictive ability in patients with CRC.

### TCGA, TIMER, and Human Cell Landscape Analyses: The Relationship Between MPO and Neutrophils in CRC

**Figure 4:**
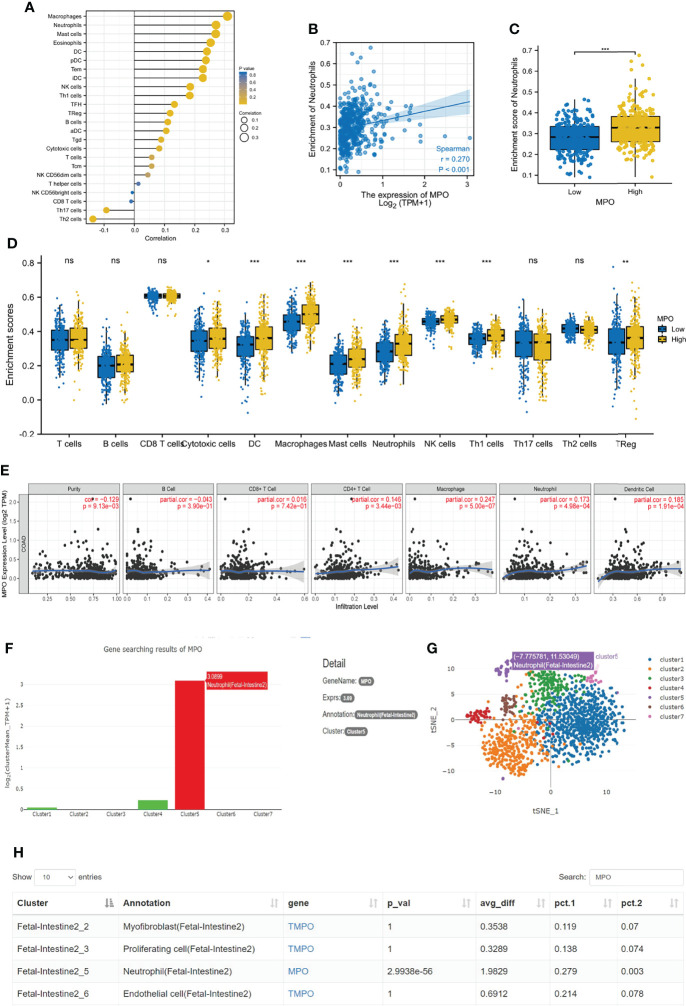
MPO was significantly correlated with the infiltration level of neutrophils in CRC. **(A)** MPO expression has a significant correlation with many immune cells infiltration. **(B, C)** The correlation between MPO and neutrophils by the Wilcoxon signed-rank and Spearman correlation tests. **(D)** We analyzed the correlation between MPO expression and T cells, B cells, CD8+ T cells, cytotoxic cells, DC, macrophages, mast cells, neutrophils, NK cells, Th1 cells, Th17 cells, Th2 cells, and Tregs using ssGSEA. **(E)** The relationship between different immune cells and MPO was analyzed in TIMER 2.0, including B cells, CD8+ T cells, CD4+ T cells, macrophages, neutrophils, and DCs. **(F–H)** The relationship between MPO and neutrophils in a single-cell RNA sequencing database. The difference was considered significant at * P < 0.05, ** P < 0.01, or *** P < 0.001. ns, no significance.

We investigated the relationship between MPO and the infiltration of different immune cells. Under the assessment, the MPO expression was demonstrated to positively correlate with the dominant immune cell type in tumors, containing macrophages, neutrophils, mast cells, eosinophils, DC, pDC, Tems, iDCs, NK cells, and other cells (**Figure 4A**). Next, we analyzed the correlation between MPO expression and T cells, B cells, CD8+ T cells, cytotoxic cells, DC, macrophages, mast cells, neutrophils, NK cells, Th1 cells, Th17 cells, Th2 cells, and Tregs using ssGSEA, which confirmed that a higher MPO expression is significantly linked with higher infiltration levels of immune cells (such as cytotoxic cells (P = 0.024), DC (P < 0.001), macrophages (P < 0.001), mast cells (P < 0.001) neutrophils (P < 0.001), NK cells (P < 0.001), Th1 cells (P < 0.001), and Tregs (P=0.008)) (**Figure 4D**). Considering that neutrophils are a vital part of nonspecific immunity and play a significant role during the process of pro- and antitumor immunity, we next performed Spearman correlation and Wilcoxon signed-rank tests to investigate the correlation between MPO expression and neutrophils, which proved that a higher MPO expression was linked with higher infiltration levels of neutrophils (**Figures 4B, C**). The TIMER database analysis among six types of immune cells showed that the expression of MPO was significantly correlated with the infiltration level of neutrophils (r = 0.173, P = 4.98e-4), macrophages (r = 0.247, P = 5.00e-7), DCs (r = 0.185, P = 1.91e-4), and CD4+ T cells (r = 0.146, P = 3.44e-3), but not with B cells and CD8+ T cells (**Figure 4E**). Further evaluation of the relationship between MPO and neutrophils was conducted using the data analyzed in a single-cell RNA sequencing database, Human Cell Landscape (http://bis.zju.edu.cn/HCL/). The results revealed that MPO was highly expressed at the single neutrophil level in the fetal intestine (**Figures 4F–H**). Taken together, MPO levels were significantly correlated with neutrophil infiltration in CRC.

#### Correlation Between MPO in CRC and Peripheral Neutrophil Counts

**Figure 5:**
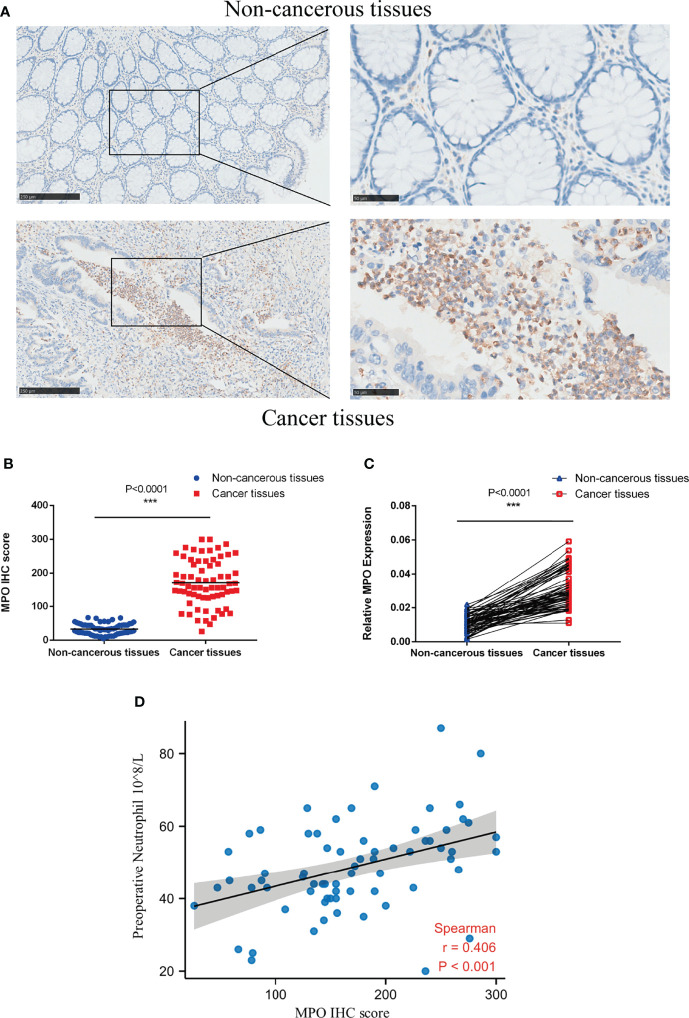
MPO was upregulated in CRC and positively correlated with peripheral neutrophil counts. **(A)** IHC was used to detect MPO protein expression in CRC and non-cancerous tissues (69 pairs); left, scale bar = 250 µm; right, scale bar = 50 µm. **(B)** Quantitative analysis of MPO IHC scores in CRC and non-cancerous tissues (69 pairs). **(C)** qPCR was used to evaluate the relative expression of MPO mRNA in CRC and adjacent non-cancerous tissues (69 pairs). **(D)** The relationship between MPO levels in CRC and peripheral neutrophil counts was determined using Spearman’s correlation test (r = 0.406, P < 0.001, n = 69). Differences were considered significant at * P < 0.05, ** P < 0.01, or *** P < 0.001.

To investigate the expression of MPO in CRC, we performed immunohistochemical staining in 69 pairs of CRC tissues and their matched adjacent non-cancerous tissues. MPO is mainly expressed in the lysosome, vesicles, and; therefore nucleoplasm. Tumor tissue is inflamed and granulocyte-rich as expected, the immunohistochemical positivity of MPO is much higher in tumor tissues than that in their matched adjacent non-cancerous tissues (**Figures 5A, B**). The mRNA expression data of MPO in 69 pairs of CRC tissues and their matched adjacent non-cancerous tissues followed the same pattern (**Figure 5C**).

We further examined whether there was a relationship between MPO expression in CRC and the peripheral neutrophil counts of CRC patients. Consistent with the TCGA analysis, Spearman’s bivariate correlation analysis showed positive correlations between MPO IHC staining scores and peripheral neutrophil counts (r = 0.406, P < 0.001; **Figure 5D**). Overall, the results revealed that MPO was upregulated in CRC and positively correlated with peripheral neutrophil counts, but the correlation was moderate.

### Clinical Data Validation: Kaplan–Meier Survival and Cox regression Proportional Hazard Survival for OS and DFS Between Patients With High and Low Preoperative Neutrophil Counts

**Figure 6:**
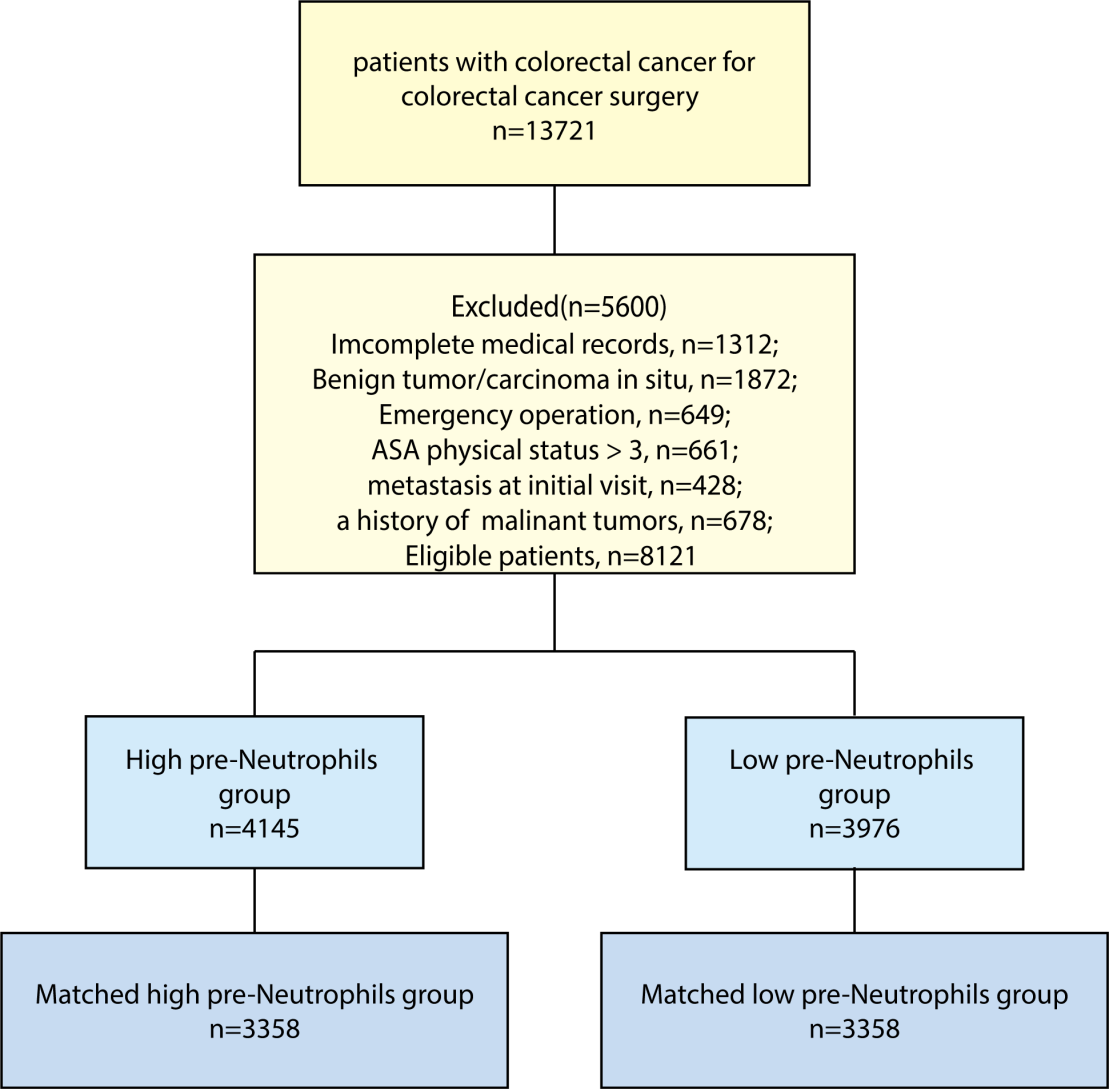
Flow chart of patient selection. Flow chart of patient selection.

A total of 8,121 patients were included in our data analysis (**Figure 6**), where the median postoperative follow-up period was 69.4 months (95% CI: 68.7–70.0). The enrolled patients were divided into two groups according to their preoperative neutrophil counts, and the cut-off value of neutrophils (3.5×10^9^/L) was calculated using an ROC curve. We observed that 51.04% (4,145 out of 8,121) of patients had high preoperative neutrophil counts. As shown in **Table 6**, higher preoperative neutrophil counts were correlated with clinicopathological characteristics, including male sex (P < 0.001), not prechemotherapy (P < 0.001), mucinous adenocarcinoma and signet-ring cell carcinoma (P < 0.001), poorer tumor differentiation (P < 0.001), more positive surgical margin (P < 0.001), advanced TNM stage (P < 0.001), more infiltrating lymph nodes > 12 (P < 0.001), more number of cancer nodules ≥ 1 (P = 0.029), more death events (P < 0.001), and more blood transfusions (P = 0.011). It showed that a high preoperative neutrophil level was likely to correlate with more malignant clinicopathological features, more blood transfusion, and poor prognosis in CRC patients.

**Table 6:** Patients’ baseline characteristics in the total study cohort and the propensity-matched cohort.

	Overall patients		P value	Matched patients		
Variables	Low pre-Neutrophils (n=3976)	High pre-Neutrophils (n=4145)		Low pre-Neutrophils (n=3358)	High pre-Neutrophils (n=3358)	P value
**Sex, n (%)**			<0.001			0.843
Male	2147 (54.0)	2665 (64.3)		1979 (58.9)	1987 (59.2)	
Female	1829 (46.0)	1480 (35.7)		1379 (41.1)	1371 (40.8)	
**Age, n (%)**			0.369			0.721
≤44	531 (13.4)	537 (13.0)		445 (13.3)	436 (13.0)	
45-54	805 (20.2)	828 (20.0)		651 (19.4)	672 (20.0)	
55-64	1453 (36.5)	1462 (35.3)		1223 (36.4)	1174 (35.0)	
65-74	836 (21.0)	910 (22.0)		721 (21.5)	745 (22.2)	
>75	351 (8.8)	480 (9.8)		318 (9.5)	331 (9.9)	
**Pre-chemotherapy, n (%)**			<0.001			0.920
No	3518 (88.5)	3929 (94.8)		3145 (93.7)	3147 (93.7)	
Yes	458 (11.5)	216 (5.2)		213 (6.3)	211 (6.3)	
**Surgical approach, n (%)**			0.406			0.366
Laparotomy	3666 (92.2)	3801 (91.7)		3102 (92.4)	3082 (91.8)	
Laparoscopy	310 (7.8)	344 (8.3)		256 (7.6)	276 (8.2)	
**Tumor histology, n (%)**			<0.001			0.658
Adenocarcinoma	3530 (88.8)	3509 (84.7)		2937 (87.5)	2913 (86.7)	
Mucinous adenocarcinoma	401 (10.1)	559 (13.5)		378 (11.3)	402 (12.0)	
Signet-ring cell carcinoma	45 (1.1)	77 (1.9)		43 (1.3)	43 (1.3)	
**Tumor differentiation, n (%)**			<0.001			0.447
Poor	726 (18.3)	955 (23.0)		672 (20.0)	719 (21.4)	
Moderate	2699 (67.9)	2788 (67.3)		2324 (69.2)	2264 (67.4)	
Well	84 (2.1)	88 (2.1)		72 (2.1)	72 (2.1)	
Unknown	467 (11.7)	314 (7.6)		290 (8.6)	303 (9.0)	
**Surgical Margin positive, n (%)**			0.001			0.759
No	3929 (98.8)	4058 (97.9)		3311 (98.6)	3308 (98.5)	
Yes	47 (1.2)	87 (2.1)		47 (1.4)	50 (1.5)	
**TNM stage, n (%)**			<0.001			<0.001
0-1	787 (19.8)	657 (15.9)		649 (19.3)	563 (16.8)	
II	1053 (26.5)	1190 (28.7)		884 (26.3)	1001 (29.8)	
III	1560 (39.2)	1592 (38.4)		1363 (40.6)	1232 (36.7)	
IV	435 (10.9)	634 (15.3)		379 (11.3)	492 (14.7)	
Unknown	141 (3.5)	72 (1.7)		83 (2.5)	70 (2.1)	
**Infiltrating Lymph nodes > 12, n (%)**			<0.001			0.093
No	970 (24.4)	826 (19.9)		733 (21.8)	677 (20.2)	
Yes	3006 (75.6)	3319 (80.1)		2625 (78.2)	2681 (79.8)	
**Number of Cancer nodule≥1, n (%)**			0.029			0.849
No	3399 (85.5)	3470 (83.8)		2827 (84.2)	2831 (84.4)	
Yes	576 (14.5)	673 (16.2)		531 (15.8)	525 (15.6)	
**Results**						
**Surgery again within 30days, n (%)**			0.800			0.514
No	3903 (98.2)	4072 (98.2)		3296 (98.2)	3303 (98.4)	
Yes	73 (1.8)	73 (1.8)		62 (1.8)	55 (1.6)	
**Death, n (%)**			<0.001			0.003
No	2957 (74.4)	2883 (69.6)		2478 (73.8)	2370 (70.6)	
Yes	1019 (25.6)	1262 (30.4)		880 (26.2)	988 (29.4)	
**Blood Transfusion, n (%)**			0.011			0.629
No	3891 (97.9)	4019 (97.0)		3276 (97.6)	3282 (97.7)	
Yes	85 (2.1)	126 (3.0)		82 (2.4)	76 (2.3)	
**Blood loss, n (%)**			0.679			0.114
<400ml	3939 (99.1)	4110 (99.2)		3323 (99.0)	3335 (99.3)	
≥400ml	37 (0.9)	35 (0.8)		35 (1.0)	23 (0.7)	

The propensity score matching was performed to reduce the imbalance due to the differences in baseline characteristics between the two groups. After matching, 3,358 pairs remained for each group. There were no significant differences in patient characteristics between the two groups in the matched cohort, except for the TNM stage (**Table 6**).

**Figure 7:**
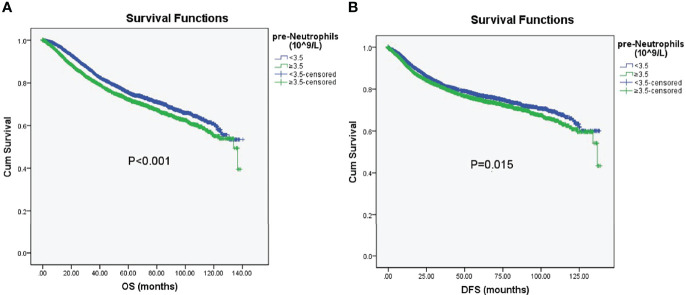
Kaplan–Meier analysis of OS and DFS. **(A)** Kaplan–Meier survival curve for OS according to preoperative neutrophils (pre-neutrophils) in the propensity score-matched cohort (OS, Logrank=13.743, P < 0.001). **(B)** Kaplan–Meier survival curve for DFS according to preoperative neutrophils (pre-neutrophils) in the propensity score-matched cohort (DFS, Logrank= 5.910, P = 0.015). Statistical significance was set at P < 0.05.

Kaplan–Meier survival analyses were further conducted to investigate patient prognosis in terms of OS and DFS after propensity score matching. The OS and DFS in the high preoperative neutrophil group were shorter than those in the low preoperative neutrophil group (OS, Logrank=13.743, P < 0.001; DFS, Logrank= 5.910, P = 0.015; **Figure 7**), demonstrating that high preoperative neutrophil levels elicited poorer prognosis in patients with CRC after matching.

After adjustment, multivariable Cox regression showed that a high preoperative neutrophil count was strongly associated with poorer OS (HR, 1.157; 95% CI, 1.055–1.268; P = 0.002) and worse DFS (HR, 1.118; 95% CI, 1.009–1.238; P = 0.033); see **Tables 7**, **8**. Other variables that significantly influenced the risk of death after multivariate analysis were older age, preoperative neoadjuvant chemotherapy, tumor differentiation, surgical margin positivity, advanced TNM stage, infiltrating lymph node > 12, number of cancer nodules ≥ 1, and blood transfusion; all of which were independent predictors of poorer OS or DFS. Overall, the preoperative neutrophil count was independently associated with increased overall mortality and cancer recurrence after CRC surgery.

**Table 7:** Univariate and multivariate Cox regression analysis for OS in the propensity-matched cohort.

Variables	Univariate HR (95% CI)	P Value	Multivariate HR (95% CI)	P Value
**Pre-Neutrophils classification**				
<3.5×10^9/L	1		1	
≥3.5×10^9/L	1.187 (1.084-1.300)	<0.001	1.157(1.055-1.268)	0.002
**Sex**				
Male	1			
Female	1.020 (0.931-1.119)	0.667		
**Age**		<0.001		<0.001
≤44	1		1	
45-54	0.936 (0.788-1.112)	0.451	1.102 (0.926-1.311)	0.276
55-64	0.964 (0.825-1.126)	0.643	1.197 (1.023-1.400)	0.025
65-74	1.221 (1.038-1.435)	0.016	1.696 (1.437-2.002)	<0.001
>75	1.910 (1.602-2.276)	<0.001	2.892 (2.416-3.460)	<0.001
**Preoperative Neoadjuvant** **chemotherapy**				
No	1		1	
Yes	1.507 (1.279-1.776)	<0.001	1.594 (1.333-1.907)	<0.001
**Surgical approach**				
Laparotomy	1			
Laparoscopy	0.911 (0.759-1.093)	0.316		
**Tumor histology**		<0.001		
Adenocarcinoma	1			
Mucinous adenocarcinoma	1.252 (1.097-1.429)	0.001		
Signet-ring cell carcinoma	2.574 (1.901-3.486)	<0.001		
**Tumor Differentiation**		<0.001		<0.001
Well	1		1	
Moderate	1.484 (1.006-2.189)	0.047	0.930(0.628-1.379)	0.719
Poor	2.986 (2.016-4.425)	<0.001	1.267(0.848-1.895)	0.248
Unknown	1.595 (1.048-2.425)	0.029	1.083(0.706-1.663)	0.715
**TNM stage**		<0.001		<0.001
0-1	1		1	
II	1.472 (1.200-1.807)	<0.001	1.398 (1.136-1.721)	0.002
III	3.250 (2.704-3.906)	<0.001	2.354 (1.939-2.857)	<0.001
IV	13.686 (11.323-16.542)	<0.001	9.819 (8.011-12.035)	<0.001
Unknown	0.701 (0.379-1.298)	0.258	0.564 (0.299-1.066)	0.078
**Surgical margin positive**				
No	1		1	
Yes	2.990 (2.302-3.885)	<0.001	1.678 (1.284-2.193)	<0.001
**Infiltrating Lymph nodes > 12**				
No	1		1	
Yes	0.718 (0.648-0.796)	<0.001	0.826 (0.741-0.920)	0.001
**Number of cancer nodule≥1**				
No	1		1	
Yes	2.911 (2.632-3.220)	<0.001	1.434 (1.284-1.601)	<0.001
**Blood Transfusion**				
No	1		1	
Yes	1.699 (1.331-2.168)	<0.001	1.420 (1.109-1.817)	0.005
**Blood loss**				
<400ml	1			
≥400ml	1.545 (1.024-2.331)	0.038		
**Surgery again within 30days**				
No	1			
Yes	1.049 (0.751-1.466)	0.778		

**Table 8:** Univariate and multivariate Cox regression analysis for DFS in the propensity-matched cohort.

Variables	UnivariateHR (95% CI)	P Value	Multivariate HR (95% CI)	P Value
**Pre-Neutrophils classification**				
<3.5×10^9/L	1		1	
≥3.5×10^9/L	1.134 (1.025-1.256)	0.015	1.118 (1.009-1.238)	0.033
**Sex**				
Male	1			
Female	0.974 (0.878-1.080)	0.616		
**Age**		<0.001		<0.001
≤44	1		1	
45-54	0.994 (0.814-1.213)	0.951	1,192 (0.976-1.457)	0.086
55-64	0.985 (0.822-1.180)	0.866	1.174 (0.978-1.409)	0.085
65-74	1.345 (1.117-1.619)	0.002	1.748 (1.448-2.111)	<0.001
>75	2.161 (1.774-2.631)	<0.001	2.877 (2.356-3.514)	<0.001
**Preoperative Neoadjuvant chemotherapy**				
No	1		1	
Yes	1.612 (1.345-1.933)	<0.001	1.892 (1.556-2.302)	<0.001
**Surgical approach**				
Laparotomy	1			
Laparoscopy	0.849 (0.689-1.045)	0.123		
**Tumor histology**		<0.001		
Adenocarcinoma	1			
Mucinous adenocarcinoma	1.292 (1.117-1.495)	0.001		
Signet-ring cell carcinoma	2.676 (1.929-3.712)	<0.001		
**Tumor Differentiation**		<0.001		<0.001
Well	1		1	
Moderate	1.381 (0.913-2.089)	0.126	0.880 (0.579-1.339)	0.551
Poor	2.718 (1.787-4.135)	<0.001	1.190 (0.774-1.830)	0.428
Unknown	1.565 (1.001-2.448)	0.050	1.056 (0.667-1.672)	0.816
**TNM stage**		<0.001		<0.001
0-1	1		1	
II	1.491 (1.215-1.830)	<0.001	1.400 (1.137-1.725)	0.002
III	3.354 (2.790-4.032)	<0.001	2.351 (1.930-2.864)	<0.001
IV	15.001 (12.099-18.598)	<0.001	9.237 (7.315-11.664)	<0.001
Unknown	0.713(0.386-1.320)	0.282	0.520 (0.274-0.986)	0.045
**Surgical Margin positive**				
No	1		1	
Yes	3.075 (2.284-4.139)	<0.001	1.578 (1.163-2.141)	0.003
**Infiltrating Lymph nodes > 12**				
No	1		1	
Yes	0.700(0.624-0.786)	<0.001	0.781 (0.693-0.880)	<0.001
**Number of cancer nodule ≥1**				
No	1		1	
Yes	2.861(2.549-3.212)	<0.001	1.449 (1.274-1.648)	<0.001
**Blood Transfusion**				
No	1		1	
Yes	1.732(1.317-2.279)	<0.001	1.562 (1.185-2.059)	0.002
**Blood loss**				
<400ml	1			
≥400ml	1.615 (1.015-2.571)	0.043		
**Surgery again within 30days**				
No	1			
Yes	0.970 (0.658-1.429)	0.876		

### The Relationship Between the MMR-Status and Neutrophil Counts, and the Effect of MMR-Status on Durvival in 668 Patients With CRC

It is well known that the type of inflammatory infiltrate is different between MMR-proficient and MMR-deficient CRCs. It may be very interesting to investigate whether this difference is reflected in peripheral neutrophil counts. Meanwhile, the MMR-status is strongly related to the prognosis in the literature. We analyzed 668 patients with MMR-status. Of these 668 patients, 307 (46.0%) patients presented with low preoperative neutrophils and 361 (54%) patients showed high preoperative neutrophils. In patients with low preoperative neutrophil, there were 16(5.2%) patients with MMR-deficient, and 291(94.8%) patients with MMR-proficient. In patients with high preoperative neutrophil, there were 32(8.9%) patients with MMR-deficient, and 329(91.1%) patients with MMR-proficient. There was no significant difference in MMR-status between two groups (P=0.068) (**Supplementary Table 1**). There was no significant difference in overall survival between MMR-deficient and MMR-proficient groups (Logrank=0.008, P=0.928) (**Supplementary Figure 2**).

## Discussion

In this study, MPO was demonstrated to be upregulated in CRC patients by evaluating the expression profile and function of MPO through TCGA, which is related to malignant progression and survival of patients with CRC. In addition, our study validated that MPO was strongly correlated with the peripheral neutrophil count of CRC patients. Furthermore, this large, retrospective study confirmed that high preoperative neutrophil count is an independent prognostic indicator for predicting OS and DFS in patients with CRC. Overall, we demonstrated that increased MPO expression was prominently correlated with a high peripheral neutrophil count, and both of these variables were independently linked with worse outcomes in CRC patients.

MPO is a cationic heme-containing peroxidase found primarily in neutrophils and minorly in monocytes (27). The bactericidal capacities of activated leukocytes have been attributed, at least partially, to the actions of MPO. MPO catalyzes the formation of reactive oxygen intermediates, including hypochlorite (HOCl), which play an important role in the killing of microorganisms (27–29); however, excessive MPO activity aggravates inflammation, leading to tissue damage. MPO is a local mediator of inflammation and also an important target for the treatment of inflammatory diseases (30); although, recent studies have shown that MPO deficiency leads to an exaggeration of the inflammatory response and affects neutrophil function, including the production of cytokines (27). For example, MPO knockout mice exhibited an enhanced response of CD4+ T cells in lymph nodes, aggravating arthritis (31). The causal link between MPO oxidation and disease is complex, and overexpression and loss of MPO expression are associated with worse outcomes. Given these contradictory reports, we were more concerned about the role of MPO in cancer. High preoperative MPO levels may improve the prognosis of postoperative CRC patients with liver metastasis (11). The high tumor infiltration of MPO-expressing cells in colorectal and breast cancers is associated with a significant improvement in prognosis (32, 33). In contrast, the MPO promoter single nucleotide polymorphism (SNP) rs2333227 enhances the malignant phenotype of CRC by regulating MPO transcriptional activity (13). Additionally, MPO overexpression in human tumors may be related to the enhancement of cell invasion and migration (14, 34). Thus, the association between MPO gene variation and CRC risk is inconsistent and warrants further investigation. In our study, bioinformatic analysis using high-throughput RNA-sequencing data from TCGA revealed that MPO expression in CRC tissues was higher than that in normal tissues. In addition, high MPO levels are associated with malignant progression. Moreover, MPO overexpression resulted in shorter OS, PFI, and DSS in this study. Therefore, MPO was validated as an independent prognostic factor for OS, PFI, and DSS based on the univariate Cox regression analysis. These findings suggest that MPO may serve as a potential prognostic marker and therapeutic target for CRC. Using IHC and qRT-PCR analyses, we confirmed that the relative expression level of MPO in CRC tissues was significantly higher than that in the matched adjacent non-cancerous tissues.

Neutrophils are one of the earliest innate immune cells to be recruited to inflammatory tissues (35). When neutrophils are activated, MPO proteins are emptied into phagosomes or secreted by degranulation. In phagosomes, MPO produces highly active hypohalogenate and nitrogen dioxide, which react easily to form different reactive oxygen species and key bactericidal and immunomodulatory products of the neutrophil MPO-halide system (36, 37). Interestingly, neutrophil aggregation and elevated levels of pro-inflammatory cytokines are associated with disease severity in patients with COVID-19, and proteomic analysis confirmed that the level of MPO in the nasopharyngeal tissue of COVID-19 patients was also increased (37). Furthermore, neutrophils treated with stimuli are rich in MPO-DNA complexes (38) and, in turn, MPO can regulate the function and immune response of neutrophils. Recent research has shown that MPO can affect the degranulation of neutrophils and improve their phagocytosis (39), and it can also “break” the migration of neutrophils and prevent their accumulation (15). The relationship between MPO in CRC and preoperative neutrophil counts is yet to be reported. In this study, we confirmed that MPO in CRC was significantly correlated with the infiltration level of neutrophils in CRC through TCGA, TIMER, and Human Cell Landscape analyses. We also validated that MPO levels in CRC positively correlated with peripheral neutrophil counts. This finding implies a potential mechanism underlying MPO and poor cancer outcomes.

Many inflammatory cells represent innate and acquired immune responses in the microenvironment of solid malignant tumors (40), where neutrophils account for a large proportion of these inflammatory cells. Tumor-associated macrophages (TAMs) can be divided into the M1 antitumor and M2 tumor-promoting phenotypes. New evidence shows that tumor-associated neutrophils (TAN) can differentiate into the N1 anti-tumor or N2 pro-tumor phenotype. Neutrophils can be transformed into a tumor-promoting state in the microenvironment (41) and, in addition to cytotoxicity, they can promote the spread of tumor cells by secreting matrix metalloproteinases and elastase to degrade the extracellular matrix. The tumor-promoting state also regulates immunosuppression by secreting reactive oxygen species and arginase-1, thus limiting T cell-dependent antitumor immunity. These tumor-promoting effects may serve as potential targets for cancer therapy (42). Neutrophils play a tumor-promoting role through the formation of neutrophil extracellular traps (NETs) within the tumor, known as NETosis (43). NETs are involved in the deterioration of a variety of diseases such as cancer, autoimmune diseases, and thrombosis (44). Most evidence shows that neutrophils can promote tumors, and the degree of neutrophil infiltration is related to poor prognosis; however, a few studies have observed that neutrophils can improve or did not affect prognosis (24, 25, 45). Our study confirmed that a higher preoperative neutrophil count was correlated with poorer OS and DFS in CRC. High preoperative neutrophil count is an independent prognostic indicator for predicting OS and DFS in patients with CRC. Yamamoto et al. also reported that a high neutrophil count was independently associated with worse survival in patients with metastatic CRC with wild-type RAS (46). Wculek et al. have suggested that the deletion of Smad4 in CRC promotes the expression of CCL15 and recruits more CCR1+ TANs and matrix metalloproteinase-9 to the metastatic site to form the premetastatic niche of disseminated tumor cells (47). Presently, the underlying mechanism behind MPO and the prognosis of patients with CRC have not yet been clarified; our research, however, suggests that neutrophils are potential key players in the mechanism linking MPO levels with poor CRC outcomes.

In our study, there was no significant difference in MMR-status between high/low preoperative neutrophil counts. There was no significant difference in overall survival between MMR-deficient and MMR-proficient patients. These results may be due the insufficient sample size. We will investigate the correlation between MMR-status and peripheral neutrophil counts in our future study. Zhu et al. reported that patients with CRC who had lost at least one MMR protein (MLH1, MSH2, MSH6, or PMS2) had a better prognosis. An unexpected finding was that there was a correlation between MMR-deficiency and elevated CD66b+ TAN levels (48). Park et al. reported that MMR-deficiency was associated with increased peritoneal involvement and poor tumor differentiation. C-reactive protein, neutrophil count, neutrophil count > 7.5×10^9^/L and NPS (neutrophil/platelet score) were higher in patients with MMR-deficient CRC than that in patients with MMR-proficient CRC before operation. In multivariate survival analysis, there was no significant correlation between MMR-deficiency and cancer-specific survival, while NPS was independently related to survival. When the analysis was limited to patients with II/III disease, NPS was still associated with survival, while MMR-status was still not associated with survival (49). Overall, the relationship between MMR-status and systemic inflammatory responses remains unclear and needs further investigation.

Our study addressed an important concern and confirmed that MPO is upregulated in patients with CRC and is related to malignant progression and survival of the patients. Furthermore, neutrophils were identified as potential key players in the mechanism linking MPO levels with poor CRC outcomes. It may be challenging to directly apply MPO in clinical settings, but the bridging role played by neutrophils can facilitate the transformation of basic research into clinical practice. One of the advantages of our clinical study is that the overall sample size (>8000) and allocation (>3000) in each group in this study were much larger than those of previous studies, and the data were obtained from one of the largest cancer centers in China. Another advantage is that the median postoperative follow-up period in this study was more than 5 years (median: 69.6 months), and we focused on the long-term outcomes of CRC patients. In addition, we used propensity score matching and multivariate Cox regression analysis to correct for confounding factors. It is usually difficult to assess the expression of MPO before operation, but the expression of neutrophils from peripheral blood can easily be determined; this aspect is the greatest contribution of our study. The prognosis of patients with CRC could be predicted using a simple and feasible method before operation. In light of this, it is suggested that anesthesiologists and surgeons pay more attention to the patients with a higher preoperative inflammation status and take measures to inhibit perioperative stress response and inflammation, so as to improve the prognosis of patients (50–52). In this regard, the present study findings can have reasonable clinical applications.

Our study had several limitations. First, the clinical validation part of the study was retrospective and non-randomized, and patient information was obtained from a single cancer center. Second, owing to various perioperative factors associated with a high neutrophil count, although we utilized propensity score matching, we still could not eliminate the potential influence of unmeasured confounders. In addition, although we found that MPO expression and preoperative neutrophil counts are involved in CRC progression, the potential mechanism needs to be further studied.

In conclusion, our study showed that increased MPO is positively correlated with high peripheral neutrophil counts, both of which serve as potential risk indicators for malignant progression and worse survival in CRC. Our study suggests that neutrophils are key players in the mechanism linking MPO levels with poor CRC outcomes, which implies the clinical applicability of our study results. In the future, well-designed prospective and basic studies are warranted to explore the underlying mechanisms further.

## Data Availability Statement

The datasets presented in this study can be found in online repositories. The names of the repository/repositories and accession number(s) can be found in the article/**Supplementary Material**.

## Ethics Statement

The studies involving human participants were reviewed and approved. This clinical retrospective study was performed at the Shanghai Cancer Center, Fudan University, Shanghai, China. The study was approved by the center’s Ethics Committee (IRB2105235-6). The patients/participants provided their written informed consent to participate in this study.

## Author Contributions

MW, and CM designed the study. MW, DW, YY, MG, and CS performed the study. MW, CZ, and QL analyzed the data. MW, DW, and YY wrote the paper. MW, MS and DZ revised the paper. All authors contributed to the article and approved the submitted version.

## Funding

This study was supported by the National Natural Science Foundation of China (No. 82002538, 82072213); Shanghai Pujiang Talent Plan (No. 2020PJD013); Clinical Research Plan of SHDC (No. SHDC2020CR1005A); and National Key Research and Development Program of China (No. 2020YFC2008400).

## Conflict of Interest

The authors declare that the research was conducted in the absence of any commercial or financial relationships that could be construed as a potential conflict of interest.

## Publisher’s Note

All claims expressed in this article are solely those of the authors and do not necessarily represent those of their affiliated organizations, or those of the publisher, the editors and the reviewers. Any product that may be evaluated in this article, or claim that may be made by its manufacturer, is not guaranteed or endorsed by the publisher.

## Supplementary Material

Supplementary Figure 1 The association between MPO and clinicopathological characteristics. **(A–J)** There was no association between MPO and clinicopathological characteristics, including T stages, N stages, history of colon polyps, colon polyps present, lymphatic invasion, perineural invasion, residual tumor, BMI, age, and sex.

Supplementary Figure 2 Kaplan-Meier analysis of OS according to MMR-status. **(A)** Kaplan-Meier survival curve for OS according to MMR-status in 668 patients with CRC. (Logrank=0.008, P = 0.928).

Supplementary Table 1 The relationship between MMR-status and high/low preoperative neutrophil counts.

## References

[1] Bray F Ferlay J Soerjomataram I Siegel RL Torre LA Jemal A. Global Cancer Statistics 2018: GLOBOCAN Estimates of Incidence and Mortality Worldwide for 36 Cancers in 185 Countries. CA Cancer J Clin (2018) 68(6):394–424. doi: 10.3322/caac.21492 30207593

[2] Chen W Zheng R Baade PD Zhang S Zeng H Bray F. Cancer Statistics in China, 2015. CA Cancer J Clin (2016) 66(2):115–32. doi: 10.3322/caac.21338 26808342

[3] Arnold M Sierra MS Laversanne M Soerjomataram I Jemal A Bray F. Global Patterns and Trends in Colorectal Cancer Incidence and Mortality. Gut (2017) 66(4):683–91. doi: 10.1136/gutjnl-2015-310912 26818619

[4] DeSantis CE Lin CC Mariotto AB Siegel RL Stein KD Kramer JL. Cancer Treatment and Survivorship Statistics, 2014. CA Cancer J Clin (2014) 64(4):252–71. doi: 10.3322/caac.21235 24890451

[5] Ndrepepa G. Myeloperoxidase - A Bridge Linking Inflammation and Oxidative Stress With Cardiovascular Disease. Clin Chim Acta; Int J Clin Chem (2019) 493:36–51. doi: 10.1016/j.cca.2019.02.022 30797769

[6] Ridzuan N John CM Sandrasaigaran P Maqbool M Liew LC Lim J. Preliminary Study on Overproduction of Reactive Oxygen Species by Neutrophils in Diabetes Mellitus. World J Diabetes (2016) 7(13):271–8. doi: 10.4239/wjd.v7.i13.271 PMC4937165 27433296

[7] Johnson RJ Klebanoff SJ Ochi RF Adler S Baker P Sparks L. Participation of the Myeloperoxidase-H2O2-Halide System in Immune Complex Nephritis. Kidney Int (1987) 32(3):342–9. doi: 10.1038/ki.1987.215 2822992

[8] Zhang C Yang J Jennings LK. Leukocyte-Derived Myeloperoxidase Amplifies High-Glucose–Induced Endothelial Dysfunction Through Interaction With High-Glucose–Stimulated, Vascular Non–Leukocyte-Derived Reactive Oxygen Species. Diabetes (2004) 53(11):2950–9. doi: 10.2337/diabetes.53.11.2950 15504976

[9] Droeser RA Mechera R Daster S Weixler B Kraljevic M Delko T. MPO Density in Primary Cancer Biopsies of Ovarian Carcinoma Enhances the Indicative Value of IL-17 for Chemosensitivity. BMC Cancer (2016) 16:639. doi: 10.1186/s12885-016-2673-7 27531373 PMC4988007

[10] Shi X Li B Yuan Y Chen L Zhang Y Yang M. The Possible Association Between the Presence of an MPO -463 G>A (Rs2333227) Polymorphism and Cervical Cancer Risk. Pathol Res Pract (2018) 214(8):1142–8. doi: 10.1016/j.prp.2018.05.018 29937309

[11] Peltonen R Hagström J Tervahartiala T Sorsa T Haglund C Isoniemi H. High Expression of MMP-9 in Primary Tumors and High Preoperative MPO in Serum Predict Improved Prognosis in Colorectal Cancer With Operable Liver Metastases. Oncology (2021) 99(3):144–60. doi: 10.1159/000510609 33027796

[12] Däster S Eppenberger-Castori S Hirt C Soysal SD Delko T Nebiker CA. Absence of Myeloperoxidase and CD8 Positive Cells in Colorectal Cancer Infiltrates Identifies Patients With Severe Prognosis. Oncoimmunology (2015) 4(12):e1050574. doi: 10.1080/2162402X.2015.1050574 26587320 PMC4635694

[13] Meng Q Wu S Wang Y Xu J Sun H Lu R. MPO Promoter Polymorphism Rs2333227 Enhances Malignant Phenotypes of Colorectal Cancer by Altering the Binding Affinity of AP-2α. Cancer Res (2018) 78(10):2760–9. doi: 10.1158/0008-5472.CAN-17-2538 PMC6084780 29540402

[14] Slattery ML Lundgreen A Welbourn B Wolff RK Corcoran C. Oxidative Balance and Colon and Rectal Cancer: Interaction of Lifestyle Factors and Genes. Mutat Res (2012) 734(1-2):30–40. doi: 10.1016/j.mrfmmm.2012.04.002 22531693 PMC3372651

[15] Rehring JF Bui TM Galán-Enríquez CS Urbanczyk JM Ren X Wiesolek HL. Released Myeloperoxidase Attenuates Neutrophil Migration and Accumulation in Inflamed Tissue. Front Immunol (2021) 12:654259. doi: 10.3389/fimmu.2021.654259 33959129 PMC8093447

[16] O'Sullivan KM Holdsworth SR. Neutrophil Extracellular Traps: A Potential Therapeutic Target in MPO-ANCA Associated Vasculitis? Front Immunol (2021) 12:635188. doi: 10.3389/fimmu.2021.635188 33790907 PMC8005609

[17] Brennan CA Garrett WS. Gut Microbiota, Inflammation, and Colorectal Cancer. Annu Rev Microbiol (2016) 70:395–411. doi: 10.1146/annurev-micro-102215-095513 27607555 PMC5541233

[18] Afghahi A Purington N Han SS Desai M Pierson E Mathur MB. Higher Absolute Lymphocyte Counts Predict Lower Mortality From Early-Stage Triple-Negative Breast Cancer. Clin Cancer Res (2018) 24(12):2851–8. doi: 10.1158/1078-0432.CCR-17-1323 PMC6366842 29581131

[19] de Nonneville A Barbolosi D Andriantsoa M El-Cheikh R Duffaud F Bertucci F. Validation of Neutrophil Count as An Algorithm-Based Predictive Factor of Progression-Free Survival in Patients With Metastatic Soft Tissue Sarcomas Treated With Trabectedin. Cancers (Basel) (2019) 11(3):432. doi: 10.3390/cancers11030432 PMC6468511 30917620

[20] Mabuchi S Matsumoto Y Isohashi F Yoshioka Y Ohashi H Morii E. Pretreatment Leukocytosis is an Indicator of Poor Prognosis in Patients With Cervical Cancer. Gynecol Oncol (2011) 122(1):25–32. doi: 10.1016/j.ygyno.2011.03.037 21514632

[21] Gouw ZAR Paul de Boer J Navran A van den Brekel MWM Sonke JJ Al-Mamgani A. Baseline Peripheral Blood Leukocytosis: Biological Marker Predicts Outcome in Oropharyngeal Cancer, Regardless of HPV-Status. Oral Oncol (2018) 78:200–6. doi: 10.1016/j.oraloncology.2018.02.003 29496051

[22] Zhang H Lv H Weng M Wang H Cata JP Chen W. Preoperative Leukocytosis is Associated With Increased Tumor-Infiltrating Neutrophil Extracellular Traps and Worse Outcomes in Esophageal Cancer. Ann Transl Med (2020) 8(7):441. doi: 10.21037/atm.2020.03.190 32395485 PMC7210211

[23] Han Y Zhao R Xu F. Neutrophil-Based Delivery Systems for Nanotherapeutics. Small (2018) 14(42):e1801674. doi: 10.1002/smll.201801674 30144279

[24] Sionov RV Assi S Gershkovitz M Sagiv JY Polyansky L Mishalian I. Isolation and Characterization of Neutrophils With Anti-Tumor Properties. J Vis Exp JoVE (2015) 100):e52933. doi: 10.3791/52933 PMC4544930 26132785

[25] Martin D Rödel F Winkelmann R Balermpas P Rödel C Fokas E. Peripheral Leukocytosis Is Inversely Correlated With Intratumoral CD8+ T-Cell Infiltration and Associated With Worse Outcome After Chemoradiotherapy in Anal Cancer. Front Immunol (2017) 8:1225. doi: 10.3389/fimmu.2017.01225 29085358 PMC5649213

[26] Tang Z Kang B Li C Chen T Zhang Z. GEPIA2: An Enhanced Web Server for Large-Scale Expression Profiling and Interactive Analysis. Nucleic Acids Res (2019) 47(W1):W556–w60. doi: 10.1093/nar/gkz430 PMC6602440 31114875

[27] Aratani Y. Myeloperoxidase: Its Role for Host Defense, Inflammation, and Neutrophil Function. Arch Biochem Biophys (2018) 640:47–52. doi: 10.1016/j.abb.2018.01.004 29336940

[28] Pattison DI Davies MJ Hawkins CL. Reactions and Reactivity of Myeloperoxidase-Derived Oxidants: Differential Biological Effects of Hypochlorous and Hypothiocyanous Acids. Free Radic Res (2012) 46(8):975–95. doi: 10.3109/10715762.2012.667566 22348603

[29] Rayner BS Love DT Hawkins CL. Comparative Reactivity of Myeloperoxidase-Derived Oxidants With Mammalian Cells. Free Radic Biol Med (2014) 71:240–55. doi: 10.1016/j.freeradbiomed.2014.03.004 24632382

[30] Masuda S Shimizu S Matsuo J Nishibata Y Kusunoki Y Hattanda F. Measurement of NET Formation *In Vitro* and *In Vivo* by Flow Cytometry. Cytom Part A (2017) 91(8):822–9. doi: 10.1002/cyto.a.23169 PMC5601186 28715618

[31] Odobasic D Kitching AR Yang Y O'Sullivan KM Muljadi RC Edgtton KL. Neutrophil Myeloperoxidase Regulates T-Cell-Driven Tissue Inflammation in Mice by Inhibiting Dendritic Cell Function. Blood (2013) 121(20):4195–204. doi: 10.1182/blood-2012-09-456483 23509155

[32] Zeindler J Angehrn F Droeser R Däster S Piscuoglio S Ng CKY. Infiltration by Myeloperoxidase-Positive Neutrophils Is an Independent Prognostic Factor in Breast Cancer. Breast Cancer Res Treat (2019) 177(3):581–9. doi: 10.1007/s10549-019-05336-3 31267330

[33] Droeser RA Hirt C Eppenberger-Castori S Zlobec I Viehl CT Frey DM. High Myeloperoxidase Positive Cell Infiltration in Colorectal Cancer is an Independent Favorable Prognostic Factor. PloS One (2013) 8(5):e64814. doi: 10.1371/journal.pone.0064814 23734221 PMC3667167

[34] Fu X Kassim SY Parks WC Heinecke JW. Hypochlorous Acid Generated by Myeloperoxidase Modifies Adjacent Tryptophan and Glycine Residues in the Catalytic Domain of Matrix Metalloproteinase-7 (Matrilysin): An Oxidative Mechanism for Restraining Proteolytic Activity During Inflammation. J Biol Chem (2003) 278(31):28403–9. doi: 10.1074/jbc.M304739200 12759346

[35] Radermecker C Hego A Delvenne P Marichal T. Identification and Quantitation of Neutrophil Extracellular Traps in Human Tissue Sections. Bio-protocol (2021) 11(18):e4159. doi: 10.21769/BioProtoc.4159 34692909 PMC8481026

[36] Tjondro HC Ugonotti J Kawahara R Chatterjee S Loke I Chen S. Hyper-Truncated Asn355- and Asn391-Glycans Modulate the Activity of Neutrophil Granule Myeloperoxidase. J Biol Chem (2021) 296:100144. doi: 10.1074/jbc.RA120.016342 33273015 PMC7857493

[37] Shrivastava S Chelluboina S Jedge P Doke P Palkar S Mishra AC. Elevated Levels of Neutrophil Activated Proteins, Alpha-Defensins (DEFA1), Calprotectin (S100A8/A9) and Myeloperoxidase (MPO) Are Associated With Disease Severity in COVID-19 Patients. Front Cell Infect Microbiol (2021) 11:751232. doi: 10.3389/fcimb.2021.751232 34746027 PMC8566808

[38] Hayden H Ibrahim N Klopf J Zagrapan B Mauracher LM Hell L. ELISA Detection of MPO-DNA Complexes in Human Plasma is Error-Prone and Yields Limited Information on Neutrophil Extracellular Traps Formed In Vivo. PLoS One (2021) 16(4):e0250265. doi: 10.1371/journal.pone.0250265 33886636 PMC8062102

[39] Fujimoto K Motowaki T Tamura N Aratani Y. Myeloperoxidase Deficiency Enhances Zymosan Phagocytosis Associated With Up-Regulation of Surface Expression of CD11b in Mouse Neutrophils. Free Radical Res (2016) 50(12):1340–9. doi: 10.1080/10715762.2016.1244821 27701922

[40] Gajewski TF Schreiber H Fu YX. Innate and Adaptive Immune Cells in the Tumor Microenvironment. Nat Immunol (2013) 14(10):1014–22. doi: 10.1038/ni.2703 PMC4118725 24048123

[41] Su H Cai T Zhang S Yan X Zhou L He Z. Identification of Hub Genes Associated With Neutrophils Infiltration in Colorectal Cancer. J Cell Mol Med (2021) 25(7):3371–80. doi: 10.1111/jcmm.16414 PMC8034475 33666342

[42] Siemińska I Poljańska E Baran J. Granulocytes and Cells of Granulocyte Origin-The Relevant Players in Colorectal Cancer. Int J Mol Sci (2021) 22(7):3801. doi: 10.3390/ijms22073801 33917620 PMC8038777

[43] Yazdani HO Roy E Comerci AJ van der Windt DJ Zhang H Huang H. Neutrophil Extracellular Traps Drive Mitochondrial Homeostasis in Tumors to Augment Growth. Cancer Res (2019) 79(21):5626–39. doi: 10.1158/0008-5472.CAN-19-0800 PMC6825588 31519688

[44] Tokuhiro T Ishikawa A Sato H Takita S Yoshikawa A Anzai R. Oxidized Phospholipids and Neutrophil Elastase Coordinately Play Critical Roles in NET Formation. Front Cell Dev Biol (2021) 9:718586. doi: 10.3389/fcell.2021.718586 34568331 PMC8458647

[45] Caruso RA Bellocco R Pagano M Bertoli G Rigoli L Inferrera C. Prognostic Value of Intratumoral Neutrophils in Advanced Gastric Carcinoma in a High-Risk Area in Northern Italy. Mod Pathol (2002) 15(8):831–7. doi: 10.1097/01.MP.0000020391.98998.6B 12181268

[46] Yamamoto T Kawada K Obama K. Inflammation-Related Biomarkers for the Prediction of Prognosis in Colorectal Cancer Patients. Int J Mol Sci (2021) 22(15):8002. doi: 10.3390/ijms22158002 34360768 PMC8348168

[47] Wculek SK Malanchi I. Neutrophils Support Lung Colonization of Metastasis-Initiating Breast Cancer Cells. Nature (2015) 528(7582):413–7. doi: 10.1038/nature16140 PMC4700594 26649828

[48] Zhu B Luo J Jiang Y Yu L Liu M Fu J. Prognostic Significance of Nomograms Integrating IL-37 Expression, Neutrophil Level, and MMR Status in Patients With Colorectal Cancer. Cancer Med (2018) 7(8):3682–94. doi: 10.1002/cam4.1663 PMC6089143 30004182

[49] Park JH Powell AG Roxburgh CS Horgan PG McMillan DC Edwards J. Mismatch Repair Status in Patients With Primary Operable Colorectal Cancer: Associations With the Local and Systemic Tumour Environment. Br J Cancer (2016) 114(5):562–70. doi: 10.1038/bjc.2016.17 PMC4782207 26859693

[50] Gatt M Khan S MacFie J. In Response to: Varadhan KK, Neal KR, Dejong CH, Fearon KC, Ljungqvist O, Lobo DN. The Enhanced Recovery After Surgery (ERAS) Pathway for Patients Undergoing Major Elective Open Colorectal Surgery: A Meta-Analysis of Randomized Controlled Trials. Clin Nutr (2010) 29:434–40. doi: 10.1016/j.clnu.2010.06.005 20116145

[51] Rothwell PM Price JF Fowkes FG Zanchetti A Roncaglioni MC Tognoni G. Short-Term Effects of Daily Aspirin on Cancer Incidence, Mortality, and Non-Vascular Death: Analysis of the Time Course of Risks and Benefits in 51 Randomised Controlled Trials. Lancet (London England) (2012) 379(9826):1602–12. doi: 10.1016/S0140-6736(11)61720-0 22440946

[52] Barron TI Connolly RM Sharp L Bennett K Visvanathan K. Beta Blockers and Breast Cancer Mortality: A Population- Based Study. J Clin Oncol (2011) 29(19):2635–44. doi: 10.1200/JCO.2010.33.5422 21632503

