# Supplementary figures and images for: Increased MPO in Colorectal Cancer Is Associated With High Peripheral Neutrophil Counts and a Poor Prognosis: A TCGA With Propensity Score-Matched Analysis
Source: Front Oncol. 2022 Jul 14;12:940706. doi: 10.3389/fonc.2022.940706 (PMC9331745; doi:10.3389/fonc.2022.940706)

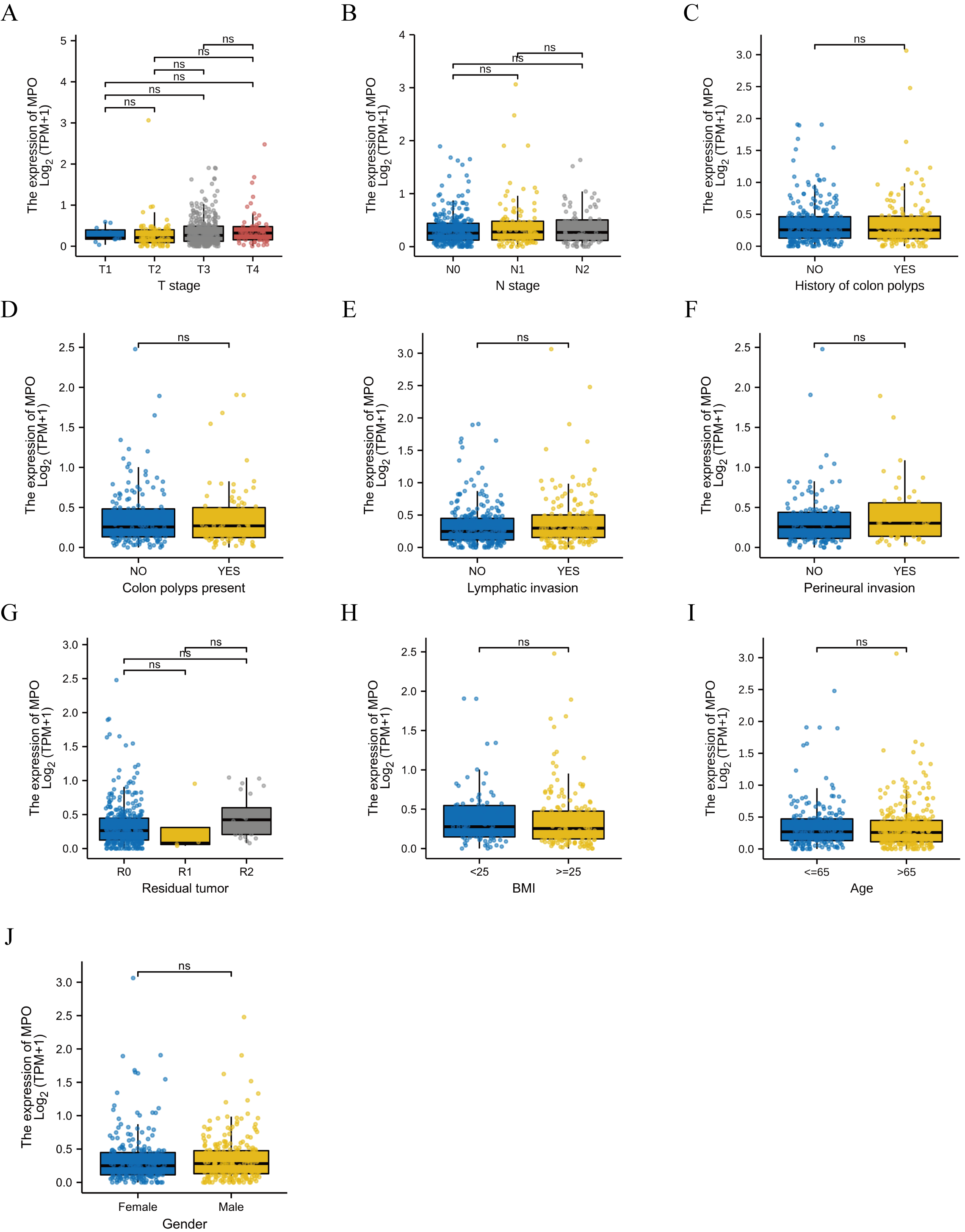

Supplement: Supplementary Figure 1 — The association between MPO and clinicopathological characteristics. (A–J) There was no association between MPO and clinicopathological characteristics, including T stages, N stages, history of colon polyps, colon polyps present, lymphatic invasion, perineural invasion, residual tumor, BMI, age, and sex. [file Image_1.tif]

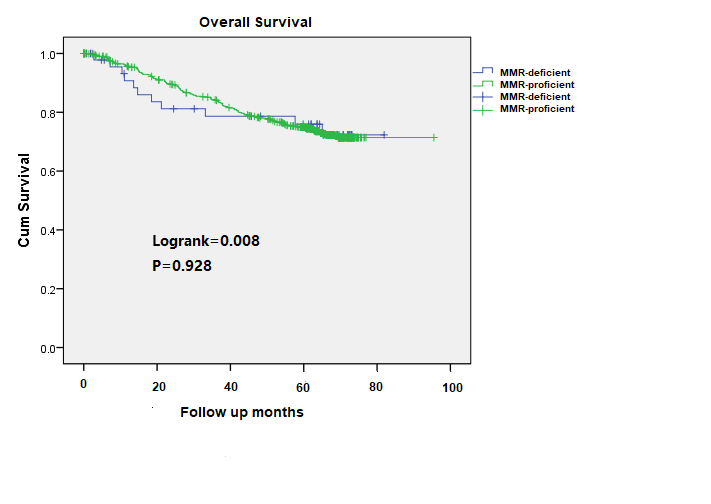

Supplement: Supplementary Figure 2 — Kaplan-Meier analysis of OS according to MMR-status. (A) Kaplan-Meier survival curve for OS according to MMR-status in 668 patients with CRC. (Logrank=0.008, P = 0.928). [file Image_2.tif]
