# Supplementary material for: Increased MPO in Colorectal Cancer Is Associated With High Peripheral Neutrophil Counts and a Poor Prognosis: A TCGA With Propensity Score-Matched Analysis
Source: Front Oncol. 2022 Jul 14;12:940706. doi: 10.3389/fonc.2022.940706 (PMC9331745; doi:10.3389/fonc.2022.940706)
Supplement: Supplementary Table 1 — The relationship between MMR-status and high/low preoperative neutrophil counts. [file Table_1.docx]

**Supplementary Table 1. The relationship between MMR-status and high/low preoperative neutrophil counts.**

| Variables | Low pre-Neutrophils  (n=307) | High pre-Neutrophils  (n=361) | P value |
| --- | --- | --- | --- |
| **MMR-status, n(%)** |  |  | 0.068 |
| MMR-deficient | 16(5.2) | 32(8.9) |  |
| MMR-proficient | 291(94.8) | 329(91.1) |  |
